# Acceptability of Using Electronic Vending Machines to Deliver Oral Rapid HIV Self-Testing Kits: A Qualitative Study

**DOI:** 10.1371/journal.pone.0103790

**Published:** 2014-07-30

**Authors:** Sean D. Young, Joseph Daniels, ChingChe J. Chiu, Robert K. Bolan, Risa P. Flynn, Justin Kwok, Jeffrey D. Klausner

**Affiliations:** 1 Department of Family Medicine, University of California Los Angeles, Los Angeles, California, United States of America; 2 Program in Global Health, University of California Los Angeles, Los Angeles, California, United States of America; 3 Los Angeles Gay and Lesbian Center, Los Angeles, California, United States of America; Rollins School of Public Health, United States of America

## Abstract

**Introduction:**

Rates of unrecognized HIV infection are significantly higher among Latino and Black men who have sex with men (MSM). Policy makers have proposed that HIV self-testing kits and new methods for delivering self-testing could improve testing uptake among minority MSM. This study sought to conduct qualitative assessments with MSM of color to determine the acceptability of using electronic vending machines to dispense HIV self-testing kits.

**Materials and Methods:**

African American and Latino MSM were recruited using a participant pool from an existing HIV prevention trial on Facebook. If participants expressed interest in using a vending machine to receive an HIV self-testing kit, they were emailed a 4-digit personal identification number (PIN) code to retrieve the test from the machine. We followed up with those who had tested to assess their willingness to participate in an interview about their experience.

**Results:**

Twelve kits were dispensed and 8 interviews were conducted. In general, participants expressed that the vending machine was an acceptable HIV test delivery method due to its novelty and convenience.

**Discussion:**

Acceptability of this delivery model for HIV testing kits was closely associated with three main factors: credibility, confidentiality, and convenience. Future research is needed to address issues, such as user-induced errors and costs, before scaling up the dispensing method.

## Introduction

After three decades of the HIV epidemic, routine HIV testing among those at highest risk remains a tremendous challenge [1], [2]. Los Angeles County currently has the second highest number of HIV cases in the US [3]. Similar to the national epidemiology of HIV infection, men who have sex with men (MSM) in Los Angeles are affected disproportionately by HIV infection; to date, male-to-male sexual behavior accounts for over 76% of all infections [4]. Barriers to testing might be particularly salient among Latino and Black MSM, as incidence rates of HIV infection are significantly higher among these populations than MSM of other racial/ethnic groups [5], [6]. Many attribute this disparity in HIV incidence to the high number of unrecognized HIV infections among Latino and Black MSM [3], [7]. Detection of HIV infection is important for individuals because knowledge of infection status has great implications in sexual risk management, forward transmission, treatment linkage, and quality of life [8].

HIV-related stigma and homophobia are factors that reduce risk perception and willingness to test for HIV [9]–[11]. HIV-related stigma is especially prevalent among Latino and Black MSM, in part, due to the role of religious institutions and religious beliefs among Latino and Black communities. As a result, many Latino and Black MSM avoid that potential stigma by remaining secretive about their sexual practices [12], [13], and avoiding HIV testing [14]. It is therefore imperative to develop innovative HIV testing strategies that can address the hidden epidemic of unrecognized HIV infections among MSM of color.

The recent Food and Drug Administration (FDA) approval of over-the-counter use of oral fluid HIV rapid self-tests may reduce the stigma associated with HIV testing and improve testing rates [15]. Those tests allow individuals to self-administer the oral fluid rapid test and interpret results at their own desired time or location. Moreover, HIV self-testing kits have been found to be acceptable among selected members of high-risk communities, such as male injection drug users [16] and MSM who rarely or never use condoms [17]. However, self-testing kits are typically distributed through pharmacies, clinics, and online stores. Those methods can be stigmatizing (due to the need for in-person test requests and/or payment through traceable methods, including credit cards and bank accounts) and might only be accessible during limited hours and at limited locations. In order to optimize uptake of HIV oral fluid rapid testing, a non-stigmatized (e.g., anonymous) and easily accessible delivery model is needed.

Electronic vending machines are currently used to dispense food/drink items, toiletries, small electronics, medications, and many other items. The customers can pay by cash (reducing likelihood that their purchase will be tracked) or by credit card for those items, and use of vending machines is an integral part of our everyday life. Vending machines could, therefore, be used to create a delivery model that offers individuals 24-hour anonymous access to HIV self-testing kits. Moreover, previous studies suggested that non-stigmatized items (e.g. candy) might serve as a psychological cover for stigmatized items (HIV self testing kits) to reduce stigma and to increase testing willingness [18], [19]. Vending machines have recently been evaluated as a feasible delivery model for HIV tests [20]; however, the acceptability of this model has not been assessed. This study sought to conduct qualitative assessments with participants to determine the acceptability of using vending machines to dispense HIV self-testing kits.

## Materials and Methods

### Ethics Information

The UCLA Institutional Review Board approved the study protocol.

### Methods

This study examined the usability and acceptability of an HIV testing process involving a vending machine that distributed free HIV self-test kits. The participants were African American and Latino men who have sex with men (MSM) living in Los Angeles. The vending machine was in one location, the parking lot at the Los Angeles Gay and Lesbian Center (located in Hollywood). For this pilot study, we used an electronic vending machine to dispense an Oraquick In-home HIV test.

We used a purposive sample method [21]. From November–December 2012 and September–October of 2013, we recruited individuals participating in a larger study designed to understand the use of social media on the uptake of HIV testing [22]. Participants were told about the vending machine as part of the larger study. If they were interested in using the vending machine to access and use an HIV test, they were emailed a personal identification number (PIN) code for the machine and asked to complete an interview about their experience. Participants entered the PIN code into the digital keypad of the vending machine to dispense the testing kit. Participants were offered a $10 gift certificate for completing the interview. In this manuscript, we represent participants 1–8 numerically.

The interviews took place between January to February, 2013 and in September–October of 2013, and used a semi-structured protocol. Questions covered four categories: demographics, vending machine use; HIV test kit use (even though the study was focused on vending machine distribution of tests, we included items on the testing experience because this experience might differ based on where and how an HIV test was received [19]; and participant recommendations. Participants provided oral consent to complete the interview. The interviews were conducted over freely-available teleconferencing software, audio-recorded, and transcribed by a researcher who specializes in qualitative methods. We used an open coding method to analyze the data from each of the four categories [23]. From this open coding process, we generated a set of codes that we confirmed by comparing the interview data by category, which established the major findings on usability and acceptability of the vending machine HIV testing process [21], [24]. Acceptability was measured based on the general sentiment of acceptance in using the machine, among participants who accessed a test from the machine.

## Results

### Participant demographics and Vending Machine HIV Testing Process Behaviors

All participants (5 Latino and 3 African American MSM) were over 18 years of age and had previously tested for HIV at some point in their lives before using the vending machine. All participants reported living within a 5–15 mile radius of the vending machine, except for one individual who was visiting Los Angeles from Texas.

We represent a summary of the participant vending machine and HIV test kit usage behaviors in Table 1. There were 59 codes emailed, 12 kits dispensed, and 8 users were interviewed. Half of the participants went to the vending machine in the morning, and the other half went in the afternoon. Most felt that the process of going to the vending machine was a private experience, though some participants expressed that the location might be a deterrent because it was placed in a location that was readily visible to people attending the Gay and Lesbian Center.

**Table 1:** Vending Machine and HIV Test Kit Usage Behavior.

Have you tested for HIV before?	8 Yes
	0 No
How far do you live from the vending machine site?	Range: 5–15 miles
	Mode: 5 miles
What time of day did you use the vending machine?	Morning = 4
	Afternoon = 4
Was using the vending machine a private experience?	Yes = 5
	No = 3
When did you use the test kit after receiving it?	Day of receipt = 6
	Within a week = 2
Do you feel you need to test again after using the kit?	Yes = 0
	No = 8
How much should someone pay for the test kit when buying it from a vending machine?	Range: $5–$25
	Mode: $5

### Usability

Participants expressed that the machine was generally acceptable due to its novelty and convenience. However, they emphasized the need to customize the look and location of the machine to improve usability and acceptability. As one participant stated:

I would change the vending machine a bit. It still looks like it is used for snacks. Customize it a little. Make the dispenser not so wide. –*Participant 2*

Many participants felt that the machine itself was not visually appealing and suggested the machine should be customized to become an HIV test kit vending machine. However, other participants believed that it should be integrated with products available in other vending machines such as snacks to increase the confidentiality of buying an HIV test kit from a machine. When asked where to put these vending machines, one participant suggested to “put them along the train lines, everyone uses the train…. the train station is really anonymous…use the same machine for snacks, like one rack on the chips dispenser could be these kits” (Participant 8). Confidentiality was important to the participants, and the location of test kits in busy areas within machines selling other products seemed like an acceptable means of availability.

Further, participants stated that not only look and confidentiality made the vending machine acceptable, but that customization was also essential to its usability. Although most participants had few problems using the vending machine, the one issue that affected use was that the HIV test kit got stuck and didn't dispense properly for a few participants. As one participant explained:

The directions said to punch in this code and the machine should dispense it. I followed the directions, but unfortunately the product got stuck between the glass and the shelf where it was coming from. Unfortunately there was nobody there to help. I was quite frustrated…I just left. Somehow or other I got another code, and then I went back the following week…. I was able to get the product, get out and go about my business. I think I took the test the following day. –*Participant 4*

This participant stated that there were many people nearby when he was trying to use the vending machine, but no one could help him, which made his experience uncomfortable since the vending machine had only the test kit and not another product. As a result, this participant left without a test kit when it got stuck in the machine. However, he was motivated to use the machine and returned a second time to get a test kit. This study showed that the usability of the machine affected participant preferences about using the machine. A number of participants expressed anxiety about the product getting stuck in the machine, and they valued the ability to be able get the test kit quickly to continue their daily activities. Among these participants, the machine was acceptable because it provided convenience and control of their HIV testing needs.

### Self-HIV Testing

All but two of the interviewed participants used the test kit on the day they received it, with the other two participants using it within that week. Participants used the HIV test kit in a private space such as a room in their home or car. A typical participant used it in the bathroom or bedroom, and took the test and read the results alone even if a friend was present for support. After taking the test, participants were asked if they believed they needed to go to a clinic to confirm the results, and all participants reported feeling confident with the result and not needing to receive a follow-up test. This may be explained by most participants' reporting they had not been highly sexually active prior to taking this test. Finally, participants reported that the tests should cost a median price of $5.

There was general acceptability in using the HIV test kit that was dispensed from the machine. It was acceptable because it was easy to use and allowed participants to control the schedule and confidentiality by choosing the location and time they wished to take the test and receive results.

When participants received their HIV test kit from the vending machine, all stated that they were confident that the kit was an accurate medical device and sanitary for use. Also, all the participants used the test kit. A common description of the process of using the kit to take the test is outlined here:

There was an instruction pamphlet inside that said what to do. There was a foil packet in there that you opened. There was a small vial and an applicator tip inside. The applicator was like a long Q-tip. You snapped off the applicator. Rubbed it between your check and gum. You dropped it into the vial. I think there is a solution in there. Then, you wait for 20–30 minutes. When you come back, there is an indicator to show the result where one line negative and two lines you were positive I think. –*Participant 5*

Although the wait time varied across participants, participants could recall the process of using the test kit and where they used it. Most participants took the test in their own home on the day it was dispensed or shortly after receiving the test kit. When taking the test, participants did not have challenges with the directions in collecting the sample, placing it in the solution, or in reading the results. While waiting for test results, most participants did other household activities. Few participants expressed anxiety about taking the test or waiting for the test results since they had control over where they would take their HIV test.

Additional appeal for this HIV testing process was that it allowed participants to control confidentiality and time efficiency related to the testing experience. As one participant explained, “you're not dealing with other people…. It's self-directed…you don't have to go to a clinic…[and] wait there…. It's simple and quick.” This participant was concerned about the wait time for the test and that other people might learn that he was testing. Similarly, another participant explained:

It was pretty, um, convenient, it was simple, and in some ways, it was private. If you didn't want anyone to know, you could do it at your convenience. It was something new and something I was curious about to get something from a vending machine that wasn't food. –*Participant 3*

This individual expressed a common motivation among the participants in wanting to know results at their convenience. Among participants, the testing process appeared to produce less anxiety compared to in-person testing because they didn't have to test and wait for results within a public context. The overall process of accessing a self-testing kit from a vending machine and being able to take it at home was very appealing to participants because it reduced anxiety and offered individual control. Moreover, participants perceived the process of using a vending machine to distribute HIV self-testing kits as acceptable both for themselves and for others, as when asked if they would suggest this process to family, friends or acquaintances, most participants reported being likely to do so.

## Discussion

In this qualitative study, use of vending machines to dispense oral-fluid, rapid HIV testing kits was found, in general, to be an acceptable test delivery model. All of the participants had been tested for HIV before, allowing them to be able to compare this experience to other more traditional testing experiences. In general, participants responded positively to the experience of receiving testing kits from vending machines once they had received the kits. Although most participants performed the test alone and did not inform others, all of them were interested in recommending this delivery model to others both in-person and online. We found that acceptability of this delivery model for HIV testing kits was closely associated with three main factors: credibility, confidentiality, and convenience (including lack of technical issues that might prevent obtaining the test) [25].

Participants found vending machines to be a credible source to dispense HIV tests, and other than a few technical issues, participants had limited concerns about the process. Tested individuals highlighted the ease of using the oral fluid HIV rapid test, and there was no ambiguity in interpreting the results. Participants expressed great confidence in their negative results and, despite the potential inaccuracies of self-testing results, did not feel the need to contact the community-based organization (CBO) provided on the instruction to obtain confirmatory testing or counseling.

Because of the unique cultural and medical needs of MSM [26] and concerns surrounding HIV testing-related stigma [9], [18], confidentiality concerns for individuals in marginalized communities might reduce their use of in-person HIV tests at clinics or CBOs. Even though some participants felt discomfort dealing with CBO security staff or employees, the location and the design of the vending machine were acceptable. Most participants agreed that vending machines should be placed in medical-related organizations, such as service-providing CBOs, clinics, and hospitals. However, if the machines were placed in public locations (e.g. train stations, bars or grocery stores), the machine should be designed to grant users more privacy to prevent others from being able to determine that a person using the machine was retrieving an HIV test.

Although the idea of using vending machines to dispense HIV tests is novel, participants might have found vending machines to be an acceptable delivery method because they are already familiar with such machines in their daily lives. For example, previous studies have already found vending machines to be appropriate delivery models for stigmatized items, such as condoms, tampons, and clean syringes for injecting drug users [27], [28]. In addition, use of vending machines to make self-testing kits accessible empowers participants to customize their HIV testing experience to be able to confidentially to test at their own convenience. Even though HIV and other diagnostic tests may produce anxiety [29], participants reported low levels of anxiety due to receiving results in a private setting.

To our knowledge, this is the first study to evaluate the acceptability of using vending machines to dispense HIV tests among Black and Latino MSM. However, the study has a few limitations. We interviewed a small number of participants from a highly selected population, and therefore lack broader generalizability. In addition, results uncovered technical difficulties with the vending machine (i.e. the product got stuck in the machine), and this incident may have impacted the testing and test retrieval experience. Future studies should also address the perspective of those who test positive, and to assess whether testing kits provide sufficient information and resources to help individuals to cope with the results. Future research can help address this question by interviewing participants who receive a positive test result. In this study, no participants reported testing positive using the self-testing kit. However, self-testing results are self-reported by participants, and therefore we were unable to know whether participants may have in fact tested positive and not reported this information. This poses an issue not only for research methods that use self-testing kits, but also for knowing when and how to follow up with self-testing participants and patients regarding linkage to care. In addition, our survey did not assess whether participants had called the test manufacturer’s free access line for information about testing results, counseling, and linkage to care. Future studies can assess participant reports of that service.

Finally, because this was an initial feasibility and acceptability study, we decided to place the vending machine near a local CBO who could provide HIV counseling and treatment services, if needed, and help link new positives to care. The clinic required that the vending machine be located in the back parking lot, which was only open from 9am–9pm. Although one of the benefits of using electronic vending machines to distribute HIV tests is the ability for widely-accessible testing kits, 24 hours a day, we were unable to provide participants with such unrestricted access to the machine for this study. However, after accounting for our acceptability and usability data, future studies can look at acceptability with vending machines with even greater access and proximity to clinical services.

Although our study was modest in size, our results suggest general acceptability among those who received tests from the machines. Upon looking through survey data, we learned that most of the participants who did not access the machine reported living more than 30 minutes away from the machine. Out of the participants who did access the machine, most reported finding the process acceptable. We therefore believe that using a single vending machine to distribute self-testing kits had limited “reach” (due to limited proximity to participants), but was generally acceptable among those who lived nearby and used the machine.

We found that using vending machines to dispense HIV tests was an acceptable delivery model among African American and Latino MSM. However, there are several issues we should address before further expanding the program. Recent studies have found that the OraQuick ADVANCE Rapid HIV-1/2 Antibody test performs very well but might miss persons with recent infection [30]–[32]. On account of user-induced errors, inaccuracies in test results, and the variation in the window period (the period during which an infection may not be detectable), individuals should be informed of the possibilities and consequences of an inaccurate, false positive or false negative test result. Access to easily understandable test information and educational materials might help address that concern. Secondly, low-income participants in high HIV prevalence areas, such as Los Angeles, might not be willing to pay for testing kits at the market price ($43) since they have access to free HIV testing through CBOs. While the potential of using vending machines as a delivery model for HIV tests is promising, more research is required to unfurl its full potential.
